# Complete Genome Sequence of the Polychlorinated Biphenyl-Degrading Bacterium *Pseudomonas putida* KF715 (NBRC 110667) Isolated from Biphenyl-Contaminated Soil

**DOI:** 10.1128/genomeA.01624-16

**Published:** 2017-02-16

**Authors:** Hikaru Suenaga, Atsushi Yamazoe, Akira Hosoyama, Nobutada Kimura, Jun Hirose, Takahito Watanabe, Hidehiko Fujihara, Taiki Futagami, Masatoshi Goto, Kensuke Furukawa

**Affiliations:** a Biotechnology Research Institute for Drug Discovery, National Institute of Advanced Industrial Science and Technology (AIST), Tokyo, Japan; b Biological Resource Center, National Institute of Technology and Evaluation (NITE), Tokyo, Japan; c Bioproduction Research Institute, National Institute of Advanced Industrial Science and Technology (AIST), Tsukuba, Japan; d Department of Applied Chemistry, Faculty of Engineering, University of Miyazaki, Miyazaki, Japan; e Research Institute for Sustainable Humanosphere, Kyoto University, Kyoto, Japan; f Department of Food and Nutrition, Beppu University, Beppu, Japan; g Education and Research Center for Fermentation Studies, Faculty of Agriculture, Kagoshima University, Kagoshima, Japan; h Faculty of Agriculture, Saga University, Saga, Japan

## Abstract

*Pseudomonas putida* KF715 (NBRC 110667) utilizes biphenyl as a sole source of carbon and degrades polychlorinated biphenyls (PCBs). Here, we report a complete genome sequence of the KF715 strain, which comprises a circular chromosome and four plasmids. Biphenyl catabolic genes were located on the largest plasmid, pKF715A.

## GENOME ANNOUNCEMENT

Polychlorinated biphenyls (PCBs) have been used widely for a variety of industrial purposes and have become serious environmental contaminants due to their chemical and physical stability. Biphenyl-utilizing bacteria can cometabolize PCBs into chlorobenzoic acids using biphenyl-catabolic enzymes. We have isolated 14 PCB-degrading bacterial strains (KF strains), including *Pseudomonas putida* KF715, from the soil near a biphenyl manufacturing plant in Kitakyushu, Japan, by enrichment culture with biphenyl as the sole source of carbon (1). These KF strains belong to phylogenetically distinct genera and exhibit specific growth characteristics on various aromatic compounds. The* bph* gene cluster involved in biphenyl/PCB degradation was first cloned from one of these strains, *Pseudomonas pseudoalcaligenes* KF707 (2, 3). We also revealed that a DNA region containing both the *bph* and salicylate catabolic *sal* genes (termed the *bph-sal* element) in the *P. putida* KF715 strain can be transferred frequently by conjugation to various *Pseudomonas putida* strains (4, 5). These KF strains, therefore, are a suitable model for investigating the diversity, distribution, and evolution of *bph* genes and PCB-degrading bacteria in biphenyl-contaminated soil. Here, we present the genomic features of the KF715 strain, which easily loses the biphenyl-utilizing ability when the cells are grown in nutrient medium (5, 6).

The genome sequence was determined by the National Institute of Technology and Evaluation (NITE). The draft genome was sequenced using 454 GS FLX+ (Roche) and HiSeq 1000 (Illumina) systems, with a standard fragment library and a paired-end library, respectively. The reads obtained by the two systems were assembled using Newbler version 2.8 (Roche), which generated the initial draft sequence data consisting of 282 contigs. The finishing was facilitated using two computer programs, GenoFinisher and AceFile Viewer (http://www.ige.tohoku.ac.jp/joho/gf_e/). Remaining gaps between contigs were closed using custom primer working and PCR amplification with standard Sanger technology. The genome of KF715 consists of a circular chromosome (6,583,376 bp, 61.9% G+C content) and four circular plasmids named pKF715A (483,376 bp, 57.3% G+C content), pKF715B (276,164 bp, 56.1% G+C content), pKF715C (94,696 bp, 62.3% G+C content), and pKF715D (30,071 bp, 54.4% G+C content).

The complete genome sequence of the KF715 strain annotated using the Rapid Annotations using Subsystems Technology (RAST) server (7) contains 6,854 predicted coding DNA sequences (CDSs), seven copies of rRNA gene operons, and 73 tRNA genes. KF715 has a larger genome (7.47 Mb) than those of the other completely sequenced* Pseudomonas putida* strains (5.77 to 6.36 Mb) (8). This RAST-based annotation revealed the presence of a large number of genes (*n* = 240) involved in the metabolism of aromatic compounds. The *bph-sal* element containing the biphenyl metabolism cluster *bphR1A1A2A3A4BC* is found on the largest plasmid, pKF715A. This suggests that the *bph-sal* element spread frequently by plasmid-mediated horizontal transfer between KF strains in the natural environment.

### Accession number(s).

The sequences with the annotation of KF715 have been deposited at DDBJ/EMBL/GenBank under accession numbers AP015029 (chromosome) and AP015030, AP015031, AP015032, and AP015033 (plasmids).
